# Chitosan Lactate Particles for Non-Compression Hemostasis on Hepatic Resection

**DOI:** 10.3390/polym15030656

**Published:** 2023-01-27

**Authors:** Yuhui Jiang, Xiaoxuan Tang, Tao Li, Jue Ling, Yifan Ge, Yumin Yang

**Affiliations:** 1 Medical School of Nantong University, Nantong 226001, China; 2 Key Laboratory of Neuroregeneration of Jiangsu and Ministry of Education, Co-Innovation Center of Neuroregeneration, Nantong University, Nantong 226001, China

**Keywords:** chitosan, non-compression hemostasis, biocompatibility, antibacterial, polysaccharides

## Abstract

The liver is the most complex vascular anatomy of all human organs, with extremely rich blood flow and fragile texture. Massive liver bleeding usually occurs after traumatic liver injury, causing severe systematic issues. Thus, bleeding control is critical in hindering mortality rates and complications in patients. In this study, non-compression hemostasis materials based on chitosan lactate particles (CLP) were developed for handling liver bleeding after injuries. CLP showed good blood biocompatibility and antibacterial performance against *S. aureus*. Taking advantage of the vital capacity of CLP to promote red blood cell and platelet adhesion, CLP exhibited in vivo homeostasis properties as non-compression hemostasis materials for traumatic liver injury, both in SD rats, New Zealand rabbits, or in beagles. Whereas CLP has better hemostasis than the commercial hemostatic agent Celox™.

## 1. Introduction

Liver is an organ with extreme rich blood flow and fragile texture. Clinically, massive liver bleeding during surgical or traumatic injury [1,2] happens frequently and can be life-threatening. For example, diffuse bleeding over 2000 mL with life-threatening outcomes has been reported [3]. Under physiological conditions, after hemorrhagic trauma, platelet and red blood cell adhesion occurs, which contributes to coagulation mechanisms [4]. During complex liver injuries, including liver resectioning, iatrogenic liver injury, and dominant intrahepatic blood vessel break, such self-hemostasis mechanism fails to reserve the vital requirements, external means for handling the diffuse bleeding are necessary [5].

Current methods applied under clinical circumstances, such as thermal hemostasis, electrocoagulation hemostasis, and focused ultrasound hemostasis and laser hemostasis [6,7,8]. However, these methods can damage surrounding tissues and introduces potential pathological problems to the organ. On the other hand, stitches are often not applicable for diffusive liver injury due to the nature of fragile organ texture and rich blood flow [9,10]. To date, bio-compatible materials loaded with hemostasis agents, such as thrombin/gelatin sponge, showed promising results in hemostasis for severe liver bleeding control [11,12,13]. However, they have poor biocompatibility and high immunogenicity and the major issues remain to be resolved in the biomaterial-based hemeostasis.

An ideal non-compression hemostatic agent should have the following properties: (a) good biocompatibility; (b) in vitro anti-infection properties; (c) strong liquid absorption capacity; (d) good hemostasis/coagulation ability; (e) cluster formation or membrane formation to produce physical barriers for hemostasis, etc. [14]. As a natural-origin marine polysaccharide, chitosan is the major chemical base of wide range of biomaterials. The wide range of supplied sources, low costs, good biocompatibility [15], and the ability to be adsorbed in human body makes it a welcoming material for tissue engineering [16,17,18]. Additionally, it is easy to process chitosan into different formats and, therefore, it is readily adjustable according to clinical needs. In the previous study of our research group, a peripheral nerve repair graft based on chitosan, chitin, medicinal gelatin, and PGLA had a significant effect on the regeneration of peripheral nerve defects. It has been successfully marketed for clinical use. In this study, chitosan lactate particles (CLP) were developed to be hemostatic reagents. Our results showed CLPs presented good in vivo homeostasis properties as non-compression hemostasis material with a distinctive antibacterial feature for traumatic liver injury, compared to commercially available products.

## 2. Materials and Methods

### 2.1. Chitosan

Chitosan purchased from Nantong Xingcheng Biological Products Co., Ltd., Nantong, China, derived from Alaskan snow crabs, deacetylation degree: 92.7 ± 1.5%, viscosity: 50–60 cP. Molecular weight and molecular weight distribution: Mw: (2.5 ± 0.1) × 10^5^, Mn: (2.09 ± 0.04) × 10^5^, Mw/Mn = 1.21 ± 0.03.

The viscosity was determined by using an NDJ-79 rotary viscometer (Shanghai Changji Geological Instrument Co., Ltd., Nantong, China), chitosan was dissolved in 1% acetic acid solution to a concentration of 10 mg/mL solution, and the viscosity was measured using an NDJ-79 rotary viscometer at 25 ± 2 °C.

Molecular weight and molecular weight distribution was determined by Agilent 1260 Infinity II LC (Agilent Technologies, lnc. Santa Clara, CA, USA) 3.0 g was completely dissolved in 1% acetic acid and detected by molecular exclusion chromatography.

Deacetylation level was determined by acid-base titration [19,20,21]. Briefly, 0.2 g of chitosan was dissolved in 30 mL of HCl titer (0.1 mol/L). Methyl orange-aniline blue were added as an indicator, and NaOH titer (0.1 mol/L) until the neuter pH. The calculation formula is as follows:

Deacetylation degree = (C_1_V_1_ − C_2_V_2_) × 0.016/[G (100% − W) × 0.0994] × 100%

C_1_, HCl standard solution concentration (mol/L);
C_2_, NaOH standard solution concentration (mol/L);
V_1_, drop the HCl standard solution volume (mL);
V_2_, consume NaOH standard solution volume (mL);
G, sample weight (g);
W, sample moisture content (%);
0.016, amount of amine equivalent to 1 mL of 1 mol/L HCl (g);
0.094, theoretical amine group content in chitosan.

### 2.2. Preparation of Chitosan Lactate Particles (CLP)

Chitosan lactate particles (CLP) is prepared through a protonation reaction according to the following steps: First, 10 g chitosan was immersed in 100 mL of absolute ethanol, lactic acid (75%, diluted with isopropanol, Xilong Chemical Co., Ltd. Shantou, China) was added slowly till the final ratio of lactic acid to chitosan reaches to 4:5 (*w*/*w*). The reaction mixture was stirred at room temperature for 3–4 h. The product was washed in ethanol to neutralize and dried to obtain chitosan lactate particles (CLP).

### 2.3. Particles Morphology, Size and Zeta Potential Analysis

Particles size and Zeta potential were detected and analyzed by Zetasizer Nano ZSP (Malvern Instruments Ltd., Malvern, UK) at a detector angle of 90, 25 °C. The charge or zeta potential of particles was determined by measuring their velocity while they were moving in an electrophoretic field, since particles and molecules that have a zeta potential will migrate towards an electrode if a field is applied, and their move speed was proportional to the field strength and their zeta potential.

Particle morphology was obtained by ZEISS-AX10 (Carl Zeiss AG, Oberkochen, Germany). The samples were evenly dispersed on the glass slide and the morphology was observed under dark field, 20× objective lens was used (scale bar = 50 μm).

### 2.4. Cytotoxicity

After samples were sterilized by Co-60 irradiation (15 kGy, Changzhou Atomic Hi-tech Radiation Co., Ltd., Changzhou, China). Samples were dipped at 200 ng/mL at 37 °C for 72 h. Log-phase L929 cells were seeded 24 h on 96-well plates at a density of 1 × 10^4^/mL, cells were incubated in the extract for 24 h (100 μL/well). The cytotoxicity was tested by the MTT kit (Shanghai Biyuntian Biotechnology Co., Ltd., Shanghai, China.). Absorbance was measured at 570 nm with Nanodrop (Thermo Fisher Scientific, Waltham, MA, USA).

Cell viability (%) = A_sample_/A_control_ × 100%

A_sample_: absorbance values of the cells treated with the sample extract.
A_control_: absorbance values after treatment of cells with basal medium.

### 2.5. Hemolysis Evaluation

The hemolysis rate of the materials was obtained according to Chinese NMPA guidelines. After samples were sterilized by Co-60 irradiation (15 kGy, Changzhou Atomic Hi-tech Radiation Co., Ltd., Changzhou, China). An amount of 1 mL of fresh anticoagulant rabbit blood was added with 1.25 mL of 0.9% normal saline to make diluted anticoagulant rabbit blood. Specifically, samples were prepared as saline suspension at the concentration of 5 mg/mL. 5 mL of the prepared sample are tested (*n* = 5). 5 mL of normal saline and 5 mL distilled water was tested as negative or positive control group. All the tests are pre-incubated at 37 °C for 30 min, followed by adding 100 μL diluted anticoagulant rabbit blood (44.4%) at 37 °C for 1 h. After incubation, 300 μL supernatant was put on a 96-well microplate, and the absorbance value was measured at the wavelength of 545 nm with Nanodrop (Thermo Fisher Scientific, Waltham, MA, USA).

Hemolysis rate (%) = (A_sample_ − A_negative_)/(A_positive_ − A_negative_) × 100%

A_sample_: absorbance values after sample treatment.
A_negative_: absorbance values after normal saline treatment.
A_positive_: absorbance values after distilled water treatment.

### 2.6. Antimicrobial Test

After samples were sterilized by Co-60 irradiation (15 kGy, Changzhou Atomic Hi-tech Radiation Co., Ltd., Changzhou, China), *S. aureus* dilution (5 × 10^4^ CFU of 7 mL sterile PBS) was incubated at 24 °C and 250 r/min for 18 h. Those with no added samples were used as a blank control group. After the shaking of the bacteria, the bacterial solution was diluted 10-fold. The 1 mL of diluted bacterial liquid was spread on the bottom of the dish and poured into Sand’s medium. Cultures were inverted by 37 °C for 24–48 h, and colonies were counted. Each set of experiments was performed in triplicate [22].

Kill (%) = (NC − NE)/NC × 100%

NC: the number of bacterial colonies in the control group.
NE: the number of bacterial colonies surviving in the experiment group.

### 2.7. Liquid Absorption

Samples were sterilized by Co-60 irradiation (15 kGy, Changzhou Atomic Hi-Tech Radiation Co., Ltd., Changzhou, China). After weighing the dry weight of the sample, 0.2 g samples (*n* = 5) in 8 mL solution (phosphate buffer solution, PBS/deionized water, DIW) for 2 h. The samples were separated from the solution by filtration, and each sample’s swelling weight was recorded [23].

Liquid absorption rate (%) = (W_wet_ − W_dry_)/W_dry_ × 100%

W_wet_: the wet weight of the sample.
W_dry_: the dry weight of the sample.

### 2.8. In Vitro Blood Coagulation Test

Samples were sterilized by Co-60 irradiation (15 kGy, Changzhou Atomic Hi-Tech Radiation Co., Ltd., Changzhou, China). To simulate a hemorrhagic wound, 0.4 mL of normal saline or 0.2 mL of the whole-blood sample was obtained from a healthy rabbit and was dropped on a marked surface with an area of 3.5 cm^2^ in a Petri dish. Chitosan lactate particles (CLP) (0.1 g) were carefully applied to the blood sample without spreading outside of the marked area, and the times spent completely absorbing the blood sample by the hemostatic powders were recorded. The flow ability of the samples was monitored through tiled glass slides with an angle of 45. The time at which the solidified gel remained still on the marked area was considered the clotting time. All results were obtained in triplicate from three separate experiments.

### 2.9. Red Blood Cells (RBCs) Adhesion Test

Samples were sterilized by Co-60 irradiation (15 kGy, Changzhou Atomic Hi-Tech Radiation Co., Ltd., Changzhou, China). The suspension of RBCs was obtained by centrifugation of citrated whole blood (CWB) (400 g, 10 min). CS (Chitosan powder, Nantong Xingcheng Biotechnology Co., Ltd., Nantong, China), YST (Chitosan Hemostatic powder, Shijiazhuang Yishengtang Medical Supplies Co., Ltd., Shijiazhuang, China), Celox™ (Celox hemostatic Granules, MedTrade Products Ltd., Crewe, UK) and chitosan lactate particles (CLP) were each weighed by 0.1 g for the test. A suspension of 100 μL of RBCs was added to the sample. Samples were incubated for 1 h at 37 °C. Non-adherent red blood cells were removed by washing with PBS (pH = 7.4). It was then transferred to deionized water (DIW) (4 mL) to break adherent red blood cells to release hemoglobin. At 37 °C it was incubated for 1 h. The 100 μL supernatant was removed, placed in a 96-well microplate, and its OD540 nm value (OD_hemostat_) was measured. The percentage of the adhered RBCs was calculated by the following equation [23]:

Adhered RBCs (%) = OD_hemostat_/OD_reference_ × 100%

OD_hemostat_: the OD540 nm value after the hemostat treatment.
OD_reference_: the OD540 nm value without the hemostatic agent treatment.

### 2.10. Platelets Adhesion Test

Samples were sterilized by Co-60 irradiation (15 kGy, Changzhou Atomic Hi-Tech Radiation Co., Ltd., Changzhou, China). Platelet-rich plasma (PRP) was obtained by centrifugation of the CWB (400 g, 10 min). CS, YST, Celox™, and CLP were each weighed by 0.1 g for the test. A suspension of 100 μL of PRP was added to the sample. Samples were incubated for 1 h at 37 °C. Non-adherent platelets were removed by washing with PBS (pH = 7.4). Then were soaked in 1% Triton X-100 solution to lyse platelets to release lactate dehydrogenase (LDH). LDH was determined using the LDH kit (Shanghai Biyuntian Biotechnology Co., Ltd., Shanghai, China), according to instructions. Finally, the OD490 nm value measured the supernatant absorbance value. The OD490 nm value of the solution consisting of 100 μL of PRP not exposed to the hemostatic agent was measured and used as a reference value. The percentage of adherent platelets was calculated by the following equation [23]:

Adhered platelets (%) = OD_hemostat_/OD_reference_ × 100%

OD_hemostat_: the OD490 nm value after the hemostat treatment.
OD_reference_: the OD490 nm value without the hemostatic agent treatment.

### 2.11. Hemostatic Test In Vivo

Samples were sterilized by Co-60 irradiation (15 kGy, Changzhou Atomic Hi-Tech Radiation Co., Ltd., Changzhou, China). In vivo hemostatic capacity of CLP was evaluated by a bleeding model of left inner lobe sections of rat, rabbit, and beagle livers [24,25]. YST and Celox™ were used as controls. All animal experiments were approved by the Ethics Committee of Animal experiments of Nantong University.

SD rats, New Zealand white rabbits, and beagles were anesthetized, abdominal shaving and skin prepared, opened wound, and exposed and lifted the liver. The liver injury was created by placed on the surface of pre-weighed gauze and filter paper, cutting on the liver, SD rats (3 ± 0.5 cm), New Zealand white rabbits (5 ± 0.5 cm), beagles (8 ± 0.5 cm). After wound exposure, the CLP particles (1 g SD rat, 3 g New Zealand, 15 g beagles) were scattered on the surface of wound and left for 5 min. The digital camera recorded the hemostasis status, and the total blood loss per liver was weighed. After 10 min, the particles were removed and flushed clean with normal saline.

### 2.12. Statistics and Reproducibility

All tests were processed in triplicate and similar results were acquired. Each group had at least three independent samples. Statistical analyses were performed using GraphPad Prism 8 software. Values were expressed as the means standard error of the mean (SEM). The differences between each group were performed using a two-tailed unpaired *t*-test. The ‘NS’ indicated no signifificant difference, * *p* < 0.05, ** *p* < 0.01, and *** *p* < 0.001.

## 3. Results and Discussions

### 3.1. Preparation and Characterization

**Figure 1:**
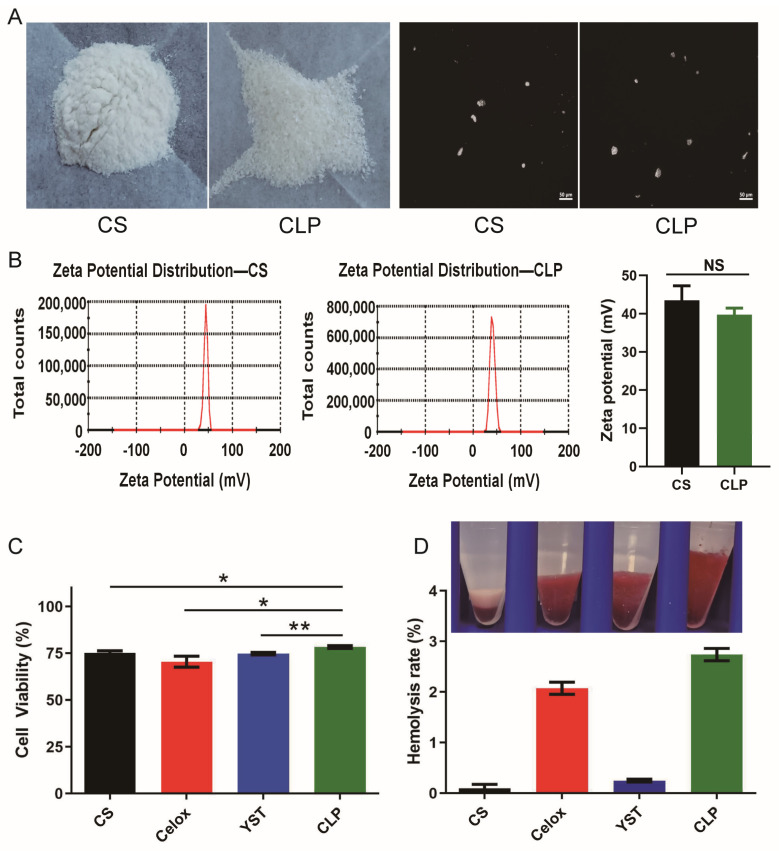
Characterization, cytotoxicity, and blood biocompatibility of hemostatic particles. (**A**) Visual image of hemostatic particles, obtained by camera and ZEISS−AX10 microscope (bar = 50 μm). (**B**) Zeta potential of hemostatic particles (*n* = 3). (**C**) Viability of L929 fibroblasts cultured with extract medium of the materials according to MTT assay (*n* = 9). (**D**) Hemolysis rate of hemostatic particles (*n* = 9). Values represent means ± SEM. The differences between each group were performed using a two−tailed unpaired *t*−test. The ‘NS’ indicated no significant difference, * *p* < 0.05, ** *p* < 0.01.

In this study, chitosan powder was successfully protonated using lactate in isopropanol as a nonaqueous medium. The amino group is protonated to have a better hydrophilicity. Positively charged chitosan lactate particles (CLP) were obtained by filtration and drying through air. Morphology of CLP was evaluated by microscopic imaging and CLP possessed a round-shaped morphology (Figure 1A). In addition, Zeta potential (surface charge) can represent the stability of the colloid through electrostatic repulsion between particles. As expected, CLP had a positive zeta potential (Figure 1B, CS: 44.4 ± 0.8 mV; CLP: 39.8 ± 1.7 mV), and the particle size of CLP was 11.3 ± 1.2 μm.

### 3.2. Biocompatibility

Biocompatibility testing is the process of observing the chemical composition of medical devices, the sterilization process, and the type of device contact for interaction with the patient’s tissue and physiological system. This testing is a critical step in the safety assessment of medical devices. Blood compatibility is mainly used to assess the safety of biomaterials in contact with blood. As a hemostatic biomaterial, chitosan lactate particles (CLP) would be applied in vivo and make contact with the blood. Therefore, the biocompatibility and blood compatibility of CLP were then evaluated. Biocompatibility of CLP was assessed by MTT assays [26], the cell survival rate in both control and CLP groups were greater than 70%, indicating the materials did not show potential cytotoxicity. Additionally, cell survival rate in CLP group was significantly higher than that in Celox™ group (Figure 1C), showing that the CLP has good biocompatibility. In addition, the blood compatibility of CLP was less than 5%, indicating that CLP had good blood compatibility (Figure 1D).

### 3.3. Anti-Infection Properties In Vitro

**Figure 2:**
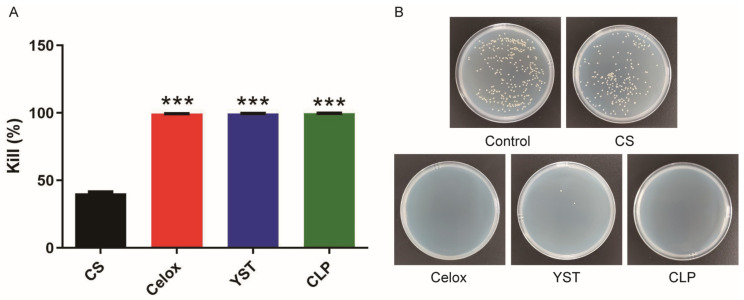
Antibacterial activity of hemostatic particles. (**A**) Antibacterial efficiency of hemostatic pacticles (*n* = 4). (**B**) Photographs of *S. aureus* formed with hemostatic particles. Values represent means ± SEM. The differences between each group were performed using two-tailed unpaired *t*-test. The ‘NS’ indicated no significant difference, *** *p* < 0.001.

Extensive wound exposure may lead to severe bacterial infection and even death, so anti-infectious properties are desired for hemostatic agents [27]. We then evaluated anti-bacterial properties of chitosan lactate particles (CLP) against Staphylococcus aureus through contact killing experiment. Our results showed that the CFU colonies of *S. aureus* incubated with the CLP was significantly lower compared to the commercially available chitosan powder (CS). This may be due to the bacteria that produced carbonate and lactic acid to create an excellent acidic environment in the CLP group, further promoting the acidation of amine group of chitosan particles, protonation of the amine group of chitosan particles for enhancing anti-infection properties. Meanwhile, Celox™ and YST also showed a good inhibitory effect on *S. aureus* (Figure 2A,B).

### 3.4. Capacity of Liquid/Blood Absorption

**Figure 3:**
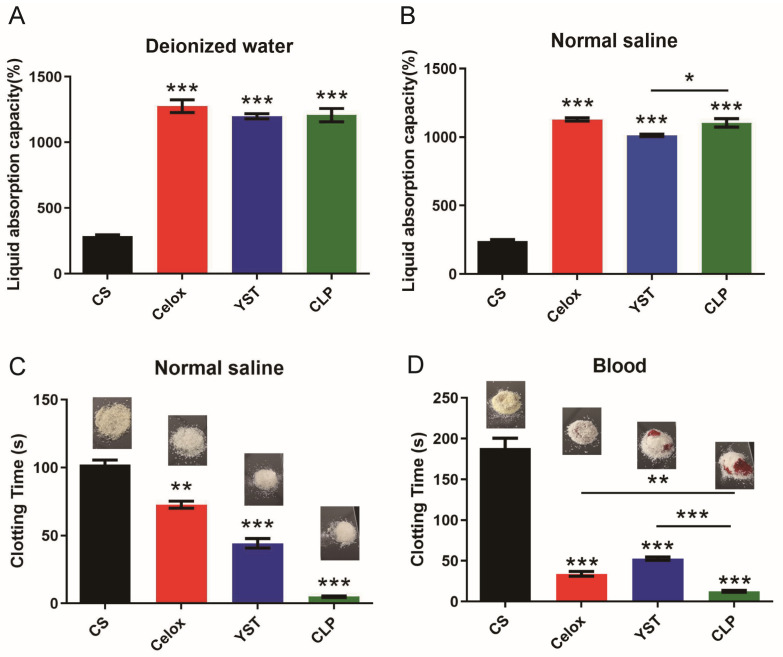
The capacity of liquid/blood absorption. (**A**) Deionized water and (**B**) Normal saline (*n* = 3). The clotting time of hemostatic particles on (**C**) Normal saline and (**D**) blood, as well as visual images (*n* = 3). Values represent means ± SEM. The differences between each group were performed using a two-tailed unpaired *t*-test. The ‘NS’ indicated no significant difference, * *p* < 0.05, ** *p* < 0.01, and *** *p* < 0.001.

The primary hemostatic mechanism of hemostatic agents is volume expansion caused by tissue fluid/blood to create mechanical compression on the bleeding site [28,29]. At the same time, a large amount of tissue fluid is absorbed, and the concentration of coagulation factors in the wound increases, accelerating coagulation. Therefore, a potent liquid absorption capacity is essential for an ideal hemostatic agent. In vitro liquid absorption of chitosan lactate particles (CLP) was evaluated in deionized water (DIW), and normal saline (NS). Our data showed that CLP exhibited a 5–6 times higher liquid absorption capacity than commercially available chitosan powder in deionized water (DIW) and normal saline (Figure 3A,B). In terms of blood absorption ability, CLP showed more rapid blood coagulation capacity than commercially available hemostatic agents, Celox™ and YST. (Figure 3C,D).

### 3.5. In Vitro Procoagulability

**Figure 4:**
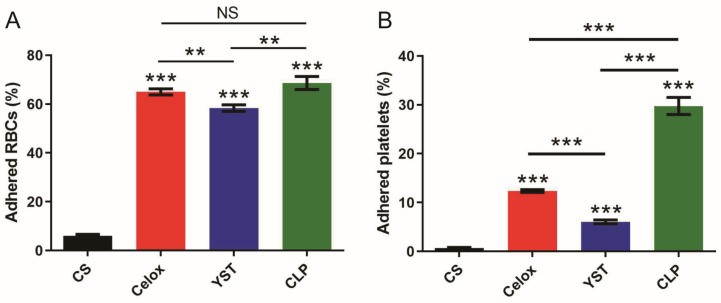
In vitro procoagulability of CLP. Ratio of adhered (**A**) RBCs and (**B**) platelets (*n* = 6). Values represent means ± SEM. The differences between each group were performed using two-tailed unpaired *t*-test. The ‘NS’ indicated no significant difference, ** *p* < 0.01, and *** *p* < 0.001.

The active coagulation cascade mainly depends on red blood cell (RBCs) aggregation and platelet adhesion [30]. When the blood vessels rupture and bleed, platelets will adhere to the bleeding site and gather with each other, forming platelet thrombosis to reduce bleeding. At the same time, they will secrete various procoagulant substances to promote blood coagulation. In addition, more coagulation factors can be absorbed in the bleeding site, forming a local blood clot, which is conducive to hemostasis. In addition, when the thrombus is formed, the platelets will further block the bleeding site through the contraction effect, and finally complete the hemostasis process. Therefore, the coagulation effects of chitosan lactate particles (CLP) were further evaluated using red blood cell and platelet adhesion assays. CLP showed a desirable RBCs adhesion capacity, comparable to Celox™ and better than YST (Figure 4A). For platelets adhesion test, CLP showed a higher platelets adhesion capacity compared to the Celox™ and YST (Figure 4B), indicating that CLP may exert a non-oppressive hemostatic effect through an active coagulation cascade. Positively charged amino groups of CLP can induce red blood cells aggregation and platelets activation to form a mechanical barrier to close the wound effectively.

### 3.6. Hemostatic Effects of CLP In Vivo

**Figure 5:**
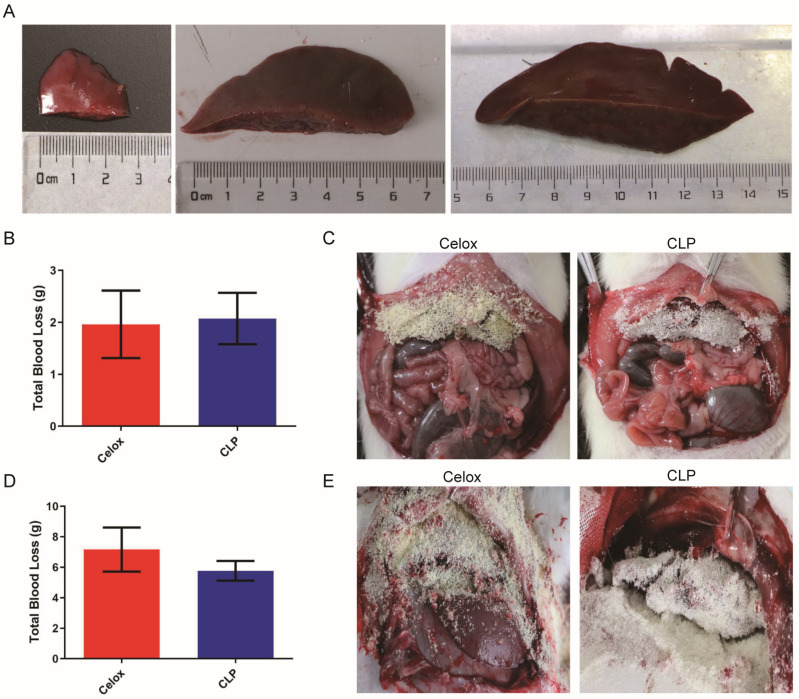

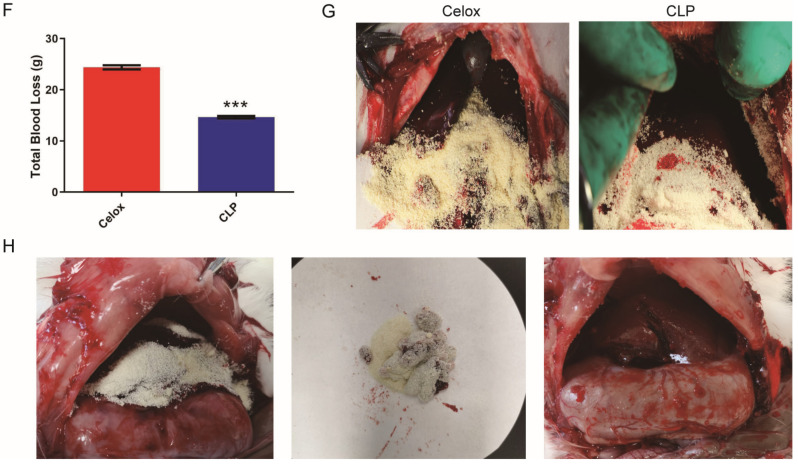
In vivo evaluation of material effectiveness on liver hemostasis. (**A**) Visual image of the removed liver (rat: 3 ± 0.5 cm; rabbit: 5 ± 0.5 cm; dog: 8 ± 0.5 cm). (**B**) Blood loss statistics, and (**C**) the hemostasis process of rat liver hemorrhage treated with these hemostatic particles. (**D**) Blood loss statistics and (**E**) hemostasis process of rabbit liver hemorrhage treated with these hemostatic particles. (**F**) Blood loss statistics and (**G**) hemostasis process of beagle liver hemorrhage treated with these hemostatic particles (*n* = 3). (**H**) Hemostasis process of rabbit liver hemorrhage treated with CLP., the stripped CLP and liver wound after normal saline washing, from left to right. Values represent means ± SEM. The differences between each group were performed using a two-tailed unpaired *t*-test. The ‘NS’ indicated no significant difference, *** *p* < 0.001.

The liver is the most complex vascular anatomy of all human organs. Hepatectomy was once considered an impossible feat, largely because of its tendency to bleed, but it is now the primary treatment for both primary and secondary liver tumors. At the same time, liver bleeding caused by war, traffic accidents, and iatrogenic injuries is also more common. Therefore, non-compression hemostasis of the liver is a clinically urgent problem. The development of ideal hemostatic agents is one of the hemostatic strategies. To further evaluate the hemostatic effect of CLP, liver resection in SD rat (3 ± 0.5 cm), New Zealand white rabbit (5 ± 0.5 cm), and beagle (8 ± 0.5 cm) models were established and treated with chitosan lactate particles (CLP) (Figure 5A). Both of Celox™ and CLP exhibited good hemostatic effects and there was no significant difference in blood loss between the Celox™ and CLP (Figure 5B–E). In the beagle hepatectomy model, there was less blood loss in CLP group than that in Celox group, which is consistent with the results of in vitro coagulation experiments (Figure 5F,G), indicating that CLP had significant advantages in non-compression hemostasis in large animals and large area hepatectomy. Moreover, 10 min after the bleed stops, the CLP was easily stripped, and the remaining particles were cleaned by flushing with normal saline (Figure 5H). When CLP reaches the bleeding wound, it increases the concentration of coagulation factors, red blood cells, and platelets at the wound opening with excellent blood absorption ability, which accelerates the coagulation reaction for rapid non-compression hemostasis.

## 4. Conclusions

The liver is the most complex vascular anatomy of all human organs, with extremely rich blood flow and fragile texture. Massive liver bleeding usually occurs after traumatic liver injury, causing severe systematic issues. Thus, bleeding control is critical in hindering mortality rates and complications in patients. In general, an ideal hemostat should have several properties, including active coagulation, strong anti-infective activity, biocompatibility, availability, low weight, and low cost. In conclusion, we developed a chitosan-based hemostatic agent via protonation of chitosan for non-compression hemostasis for large area hepatectomy. The good liquid absorption capacity of CLP facilitated the increased concentration of red blood cells and platelets at the wound sites. Positively charged amino groups in CLP electrostatically attracts RBCs and platelets and induced RBCs aggregation, accelerates platelets activation, coagulation response and formation of thrombosis at the wound region. In addition, the CLP also possessed good blood compatibility and strong in vitro anti-infection properties. Significantly, CLP exhibited excellent capacity on liver hemostasis in vivo. Therefore, we believe that the CLP have great potential in hemostasis, wound healing, and oncology fields.

## Data Availability

Data available on request due to technical limitations.
